# Endoscopic endonasal dacryocystorhinostomy learning curve

**DOI:** 10.5935/0004-2749.20220030

**Published:** 2022

**Authors:** Mustafa Vatansever, Evren Aydın, Erdem Dinç, Özer Dursun, Ömer Özer, Gülhan Örekici Temel

**Affiliations:** 1 Department of Ophthalmology, Faculty of Medicine, University of Mersin, Mersin, Turkey; 2 Department of Otorhinolaryngology, Toros State Hospital, Mersin, Turkey; 3 Department of Biostatistics and Medical Informatics, Faculty of Medicine, University of Mersin, Mersin, Turkey

**Keywords:** Lacrimal duct obstruction, Nasolacrimal duct/ sur gery, Dacryocystorhinostomy/methods, Endoscopy, Ophthalmology/education, Obstrução dos ductos lacrimais, Ducto nasolacrimal/cirurgia, Dacriocistorinostomia/métodos, Endoscopia, Oftalmologia/educação

## Abstract

**Purpose:**

To compare the learning curves of the specialists in two different fields without previous endoscopic endonasal dacryocystorhinostomy experience as well as to reveal the related complications with surgical success rates.

**Methods:**

We retrospectively investigated 90 patients who received consecutive endoscopic endonasal dacryocystorhinostomy with mucosa preservation by an ophthalmologist (Group 1, n=45) and an otorhinolaryngologist (Group 2, n=45) between October 2017 and October 2019. Patients who were admitted with epiphora complaints and diagnosed with primary acquired nasolacrimal duct obstruction through lacrimal irrigation test and aged >18 years with at least 6 months of follow-up were included in the study. In all cases, additional pathologies such as septum deviation were evaluated by performing maxillofacial imaging. Patients’ medical records were evaluated in terms of surgery duration, complications, and functional achievements.

**Results:**

The mean surgical duration of the patients in Group-2 was 36.27 ± 11.61 min, while it was 43.62 ± 16.89 min in Group-1; the difference was statistically significant (p=0.018). Functional achievements in Group 1 was 84.4% (73.3% in the first set of 15 cases, 93.3% in the last set of 15 cases) in Group 2; this rate was 88.9% (80% in the first set of 15 cases, 93.3% in the last set of 15 cases), and the difference was not statistically significant (p=0.53). Septum intervention in addition to endoscopic surgery in both the groups (p=0.03, p=0.005, respectively) and intense bleeding during surgery (for both the groups, p<0.0001) significantly decreased the functional success.

**Conclusion:**

Endoscopic endonasal dacryocystorhinostomy, performed after the necessary training, can provide high success and low complication rates when even conducted by ophthalmologists who are unfamiliar with endoscopic surgery after an experience of 30 cases.

## Abstract (pt)

**Objetivos:**

O objetivo deste estudo é comparar as curvas de aprendizagem dos especialistas em dois campos diferentes sem experiência prévia de dacriocistorrinostomia endonasal endoscópica e revelar as complicações com as taxas de sucesso cirúrgico.

**Métodos:**

Foram investigados retrospectivamente 90 pacientes que receberam dacriocistorrinostomia endonasal endoscópica consecutiva com preservação da mucosa realizada por um oftalmologista (Grupo 1, n=45) e realizada por um otorrinolaringologista (Grupo 2, n=45) entre outubro de 2017 e outubro de 2019. Foram incluídos no estudo pacientes admitidos com epífora e diagnosticados com obstrução primária do ducto nasolacrimal adquirido como resultado do teste de irrigação lacrimal, com idade superior a 18 anos e com, pelo menos, 6 meses de acompanhamento. Em todos os casos, patologias adicionais, como o desvio do septo, foram avaliadas por meio da realização de imagens maxilofaciais. Os prontuários dos pacientes foram avaliados quanto à duração da cirurgia, complicações e desempenho funcional.

**Resultados:**

A média de duração cirúrgica dos pacientes no Grupo-2 foi de 36,27 ± 11,61 minutos, enquanto no Grupo-1 foi de 43,62 ± 16,89 minutos, sendo a diferença estatisticamente significativa (p=0,018). O desempenho funcional no Grupo 1 foi de 84,4% (73,3% nos primeiros 15 casos, 93,3% nos últimos 15 casos) no Grupo 2, essa taxa foi de 88,9% (80% nos primeiros 15 casos, 93,3% nos últimos 15 casos) e a diferença não foi estatisticamente significativa (p=0,53). A intervenção do septo além da cirurgia endoscópica em ambos os grupos (p=0,03, p=0,005, respectivamente) e sangramento intenso durante a cirurgia (para ambos os grupos, p<0,0001) diminuiu significativamente o sucesso funcional.

**Conclusão:**

A dacriocistorrinostomia endonasal endoscópica, realizada após o treinamento necessário, pode ser realizada com alto sucesso e com baixas taxas de complicações por oftalmologistas que não estão familiarizados com a cirurgia endoscópica após adquirirem experiência com trinta casos.

## INTRODUCTION

Primary acquired nasolacrimal duct obstruction (PANDO) is a clinical condition characterized by epiphora and acute and chronic dacryocystitis and the only treatment option known is surgery. External dacryocystorhinostomy (EX-DCR) was first described by Toti and remains the gold standard treatment method that has survived despite various modifications^(1)^. On the other hand, the endonasal dacryocystorhinostomy (DCR) method defined by Caldwell before Toti did not attract much attention due to its high failure rates^(2)^. However, parallel to the advancements made in the development of instruments used in endonasal surgery with fiber optic imaging, the success of this surgery has increased and has started to attract more attention in the recent years^(2-4)^. The endoscopic procedure offers important advantages, such as no skin incision, no damage to the lacrimal pump system, less intraoperative bleeding, and less postoperative morbidity^(4-7)^. In addition, one of the most important causes of failure is the closure of the ostium created due to postoperative granulation and synechia^(8)^. For this purpose, maintaining the bone window wide and using mucosal flaps played an important role in increasing the surgical success^(9,10)^. In a study conducted by Dolman, the endonasal DCR success rate was reported to be 90.2%, while this rate was 89.1% for EX-DCR^(11)^. Despite all these advantages, the learning process of endoscopic endonasal DCR (EE-DCR) covers a longer period than EX-DCR^(12)^. The main reason for this is the adaptation process to the optimal use of endoscopes and surgical instruments used in the imaging of this region. In the EX-DCR, the learning process is faster since there is no use of these equipment. Especially for ophthalmologists, this process may be longer and more complicated due to the unfamiliar anatomy, the use of monocular imaging method not used in other surgeries, and surgical instruments^(13)^.

The aim of the present study was to evaluate the learning curves of two specialists in a comparative field without any previous EE-DCR experience as well as to reveal the complications with surgical success rates. Meanwhile, we intended to determine the optimal number required for successful endoscopic endonasal DCR.

## METHODS

The required ethical permissions were obtained from the Mersin University Clinical Research Ethics Committee prior to the presented study. Informed consent form was obtained from all patients included. The study was conducted in compliance with the Declaration of Helsinki. In our retrospectively designed study, the data obtained from the patients’ medical records were examined. Between October 2017 and October 2019, 90 patients who were consecutively applied with EE-DCR and mucosa preservation by an ophthalmologist (Group 1, n=45) and otorhinolaryngologist (Group 2, n=45) working at the Toros State Hospital were included in the study. All surgeries were accompanied by an experienced surgeon observer on EE-DCR. Patients aged >18 years who had applied to the epiphora with a diagnosis of PANDO as a result of the lacrimal irrigation test and had a follow-up of at least 6 months were included in the study. Patients with previous DCR surgery, a history of maxillofacial trauma or canalicular stenosis, and diagnosis of secondary dacryostenosis were excluded. Preoperative endoscopic examination of the nasal cavity was performed in all patients. In addition, maxillofacial imaging was performed in the necessary cases. The patients’ records were evaluated in terms of surgery duration, complications, and functional achievement.

### Surgical technique

**Figure 1:**
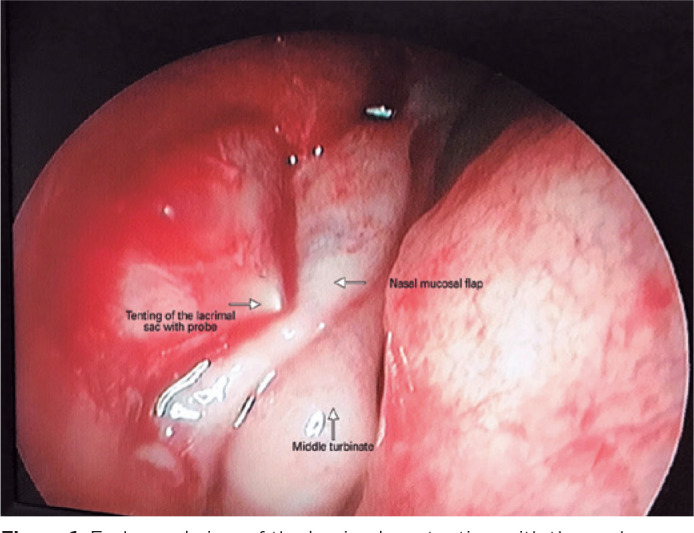
Endonasal view of the lacrimal sac tenting with the probe.

All surgeries were performed under general anesthesia, and intranasal adrenaline-soaked cotton was applied before surgery to provide decongestion and vasoconstriction. Then, a mixture of lidocaine and epinephrine was applied submucosally below the maxillary line up to the front of the middle turbinate axilla. The 0° rigid endoscope (Storz, St Louis, MI, USA) was used for imaging purposes. The mucosal incision, which started with the MVR knife from the upper part of the inferior turbinate in order to prevent the possible bleeding from preventing the image, was continued curve lineer to superior after extending approximately 10 mm towards the anterior. It was extended to 5-6-mm above the sticking point of the middle turbinate and terminated at the posterior end. The mucosal flap, which was created using freer periosteum elevator, was tilted toward the posterior. Thus, only one posterior-based nasal mucosa flap was created. With the help of Kerrison punch, the bone tissues were removed and a diamond burr was used whenever necessary. After the lacrimal sac mucosa was exposed, the lacrimal sac mucosa was tented by using a probe of the silicone tube (Silicone tube, FCI Ophthalmics, Marshfield Hills, U.S), and lacrimal sac flaps were created with the help of an MVR knife (Figure 1). After intubation with silicone tubes, the nasal mucosa flap was extended into the lacrimal sac. To stabilize these flaps and minimize synechiae, a gelatin sponge (Spongostan Standart, Ethicon, Istanbul, Turkey) soaked with steroids was placed over the nasal mucosa and lacrimal sac flaps. Endoscopic septoplasty was performed in cases that prevented visualization of the nasal cavity and narrowed the surgical area by an otolaryngologist. All patients used topical drops containing 0.3% tobramycin and 0.1% dexamethasone (Tobradex, Alcon) 4-times a day for 2 weeks. Saline nasal spray was used for a week to flush out the debris from the surgical site. As an oral antibiotic, 1-g amoxicillin/clavulanate potassium tablets (Augmentin, GlaxoSmithKline, Istanbul, Turkey) were used twice a day for 1 week. The patients were followed up on the 1st, 3rd, and 7th days of the postoperative week, once a week for the first month, and then once a month for 6 months. Endoscopic nasal cavity examination was performed on all patients at each visit. Any granulation tissues or fibrous tissues on the ostium was removed. Silicone tubes placed during the surgery were removed after 3 months. Surgical success was defined based on endoscopic examination of methylene blue dropped on the conjunctiva fornix surface to reach the rhinostomy area. Surgical failure was defined as no improvement in the epiphora and/or absence of passing methylene blue dropped into the conjunctival fornix on endoscopic examination.

### Statistical analysis

The numbers and percentages for the categorical data, and the mean and standard deviation values for the continuous structure were calculated. Relations between categorical data variables were examined with Chi-square test. While the student’s *t*-test was used to examine whether there was a difference between the 2 groups’ average values, and the z test was used to examine whether there was a difference between the two ratios. Statistical significance was considered as *p*<0.05. The program used for statistical analysis was TIBCO Statistica^®^.

## RESULTS

The average ages of the case patients in Group 1 was 54.16 ± 11.75 years and in Group 2 was 52.53 ± 16.64 years; the difference in the age was statistically insignificant (p=0.59). Of the 45 patients in Group 1, 14 (31.1%) were men and 31 (68.9%) were women. Similarly, 14 (31.1%) of the patients in Group 2 were men and 31 (68.9%) were women. The mean duration of the follow-up examination was 11.26-2.72 months in Group 1 versus 11.22-2.74 months in Group 2. The gender distribution and follow-up time in both the groups were similar, and the difference was not significant (p=0.61, p=0.93, respectively). The mean surgical time of the patients in Group 2 was 36.27 ± 11.61 min; this time was 43.62 ± 16.89 min in Group 1, and the difference was statistically significant (p=0.018).

The functional success rate in Group 1 was 84.4%, while it was 88.9% in Group 2; the difference was not statistically significant (p=0.53). Septoplasty was performed for 16 (35.5%) patients in Group 1 and 11 (24.4%) in Group 2. No statistically significant difference was noted between the groups in terms of patients who underwent septoplasty (p=0.16). Septoplasty was performed in 7 patients in the first 15 cases of both the surgeons. In the last 15 cases, septoplasty was performed in 2 cases of ENT and 4 cases of ophthalmologist. When the last 15 cases were compared with the first 15 ones, the rate of septoplasty was found to have decreased (p=0.04). In addition to endonasal surgery in both the groups, septum intervention (p=0.03, p=0.005, respectively) and intraoperative intense bleeding (p<0.0001 for both the groups) were found to significantly reduce the functional achievement. Intraoperative intense bleeding was defined as bleeding that disrupted the endoscopic imaging, prevented the course of surgery, and required hemostasis. Three patients in Group 1 had post-operative ecchymosis and 1 patient had post-operative hemorrhage. In Group 2, 2 patients had ecchymosis and 1 patient had post-operative hemorrhage. Cerebrospinal fluid leakage or orbital fat exposure was not observed in any of the patients. The total postoperative complication rate was 8.8% for Group 1 and 6.6% for Group 2. When Group 1 and Group 2 were compared in terms of postoperative complications, the difference was not found to be statistically significant.

When the two groups were compared in terms of the first and second 15 cases, the mean duration of surgery was found to be significantly lower in Group 2 (for both the parameters, p<0.0001). However, when compared with the last 15 cases, the difference was statistically insignificant (p=0.64) (Table 1). When the two groups were compared in terms of the first, second, and third 15 cases, intensive bleeding, the use of rounger, the use of burr, and the functional results were similar, with no statistical difference between the two groups (Table 1).

**Table 1:** Surgical duration, bleeding, Rounger and DRILL usage rates, and the distribution of functional achievements in both the groups

	Group-1 1^st^-15^th^ case	Group-2 1^st^-15^th^ case	p	Group-1 16^th^-30^th^ case	Group-2 16th-30th case	p	Group-1 31^st^-45^th^ case	Group-2 31^st^-45^th^ case	p
Surgery duration (min)	62.80 ± 9.47	49.67 ± 7.28	<0.0001	43.67 ± 3.59	35.13 ± 3.06	<0.0001	24.40 ± 2.19	24 ± 2.50	0.64
Intense haemorrhage	5 (%33.3)	7 (%46.7)	0.45	3 (%20)	3 (%20)	1.0	2 (%13.3)	2 (%13.3)	1.0
Use of Rounger	11 (%73.3)	9 (%60)	0.43	12 (%80)	13 (%86.7)	0.62	13 (%86.7)	13 (%86.7)	1.0
Use of DRILL	4 (%26.7)	6 (%40)	0.43	3 (%20)	2 (%13.3)	0.62	2 (%13.3)	2 (%13.3)	1.0
Functional achievement	11 (%73.3)	12 (%80)	0.66	13 (%86.7)	14 (%93.3)	0.53	14 (%93.3)	14 (%93.3)	1.0

In Group 1, the duration of surgery in the first 15 cases and the duration of surgery in the third 15 cases were found to be significantly shorter (p<0.0001). Similarly, it was observed that the surgical time in Group 2 was shortened in the third set of 15 cases (p<0.0001). Simultaneously, there was no significant difference between the case slices in Group 1 in terms of intense bleeding, the use of rounger, the use of burr, and the functional results (p=0.19, p=0.35, p=0.35, and p=0.13, respectively). In Group 2, the frequency of intense bleeding in the third set of 15 cases was found to be significantly lower than that in the first set of 15 cases (p=0.042), but there were no significant differences in other parameters.

## DISCUSSION

Although the EE-DCR was first described in 1893, its popularity started to rise since the 1990s. Especially, the development of intranasal imaging and the use of fiber optic lighting facilitated the manipulations of surgical instruments and provided a better understanding of the anatomy over a period of time, which together increased the success rate of this surgery^(5)^. Although the EX-DCR is the gold standard with extremely high success rate, EE-DCR is more preferred due to its success rates approaching those of the classical surgery as well as its extremely important advantages^(5)^, such as no skin incision, no damage to the lacrimal pump system, less intrao perative bleeding, and less postoperative morbidity^(4,5)^. However, the most important disadvantage of endonasal surgery is that the learning curve is steeper than that in the classical surgery. For ophthalmologists, the main reasons for the learning curve being steeper than that in classical surgery is the lack of knowledge of the anatomy of the region, the need for bimanual surgery due to the need for endoscope use, the inexperience in this regard, the use of unfamiliar surgical instruments, and the application of the operation with a two-dimensional image. Several of these factors, which are considered for ophthalmologists, do not constitute an important obstacle for otorhinolaryngologists. However, inadequate domination of the lacrimal system anatomy comes to the fore as well. In the present study, the learning curve of an ophthalmologist who was trained in this field but had no surgical experience was evaluated and compared with that of an otorhinolaryngologist. Unlike past studies in the literature, the learning curves of the experts in two different fields were evaluated for themselves but also comparatively.

Önerci et al. reported that the success rate in endonasal surgery performed by experienced surgeons was 94%, while it was 58% in surgeries performed by inexperienced surgeons^(14)^. In another study, functional achievements were reported as 95% for experienced surgeons and 89% for inexperienced surgeons^(15)^. In this study, while the average functional achievement in Group 1 was 84.4%, this rate was 88.9% in Group 2, and the rates obtained seemed lower than those reported in the literature. Nevertheless, when the functional achievement of the patients in the third set of 15 cases in both the groups was analyzed, the value came to 93.3%. This rate seems to be compatible with that reported in the literature. In addition, as expected in both the groups, parallel to the increase in experience, the functional achievement increased as well to reach the data reported in the literature. Concurrently, when the surgeries performed in the first set of 15 cases were evaluated, the functional achievements were found to be above the success rates of inexperienced surgeons as cited by Önerci et al.^(14)^. The main reason for this may be the advancements in the imaging technology, surgical equipment, and surgical techniques.

Past studies have reported that 10-46% of all cases undergoing EE-DCR require septoplasty^(16)^. The main reason for this is the difficulty of septum deviation in endoscopic imaging and manipulations. In this study, 30% of all patients underwent septum intervention with endonasal DCR; this result is consistent with that in the literature. Considering that septum intervention performed during surgery facilitates the EE-DCR procedure, it may have a negative effect on the functional success^(17)^. In this study, in the first set of cases, the need for septoplasty was more, and it decreased with increasing level of experience. Moreover, it was found that septoplasty significantly reduced the functional achievements in both the groups. One of the studies supporting this result by Malhotra et al. emphasizes the importance of septoplasty for inexperienced surgeons in the learning curve^(18,19)^.

One of the important factors that decrease the functional achievement after endonasal surgery is bleeding during surgery^(19,20)^. In this study, a decrease in the number of cases with bleeding during surgery was observed with an increase in the surgical experience in both the groups. Similar to that in the studies in the literature, the functional achievements in cases with bleeding decreased significantly in the present study. Bleeding during surgery can be prevented by providing better local decongestion, less damage to the surrounding tissues with increased experiences, changes in the patient position, and hypotensive anesthesia. Herzullah et al. compared burr and kerrison with the rounger and found no difference in terms of the surgical success; the use of burr prolonged the duration of surgery and increased the risk of thermal damage as the operating temperature reached 70°C^(20)^. Moreover, the use of burr involved the risk of orbital fat tissue prolapse due to orbital wall damage and the leakage of cerebrospinal fluid as a result of ethmoid sinus or skull base penetration^(21)^. In this study, as the experience level of the surgeon increased, the use of burr decreased, which avoided the development of any related complications.

When the surgical duration in both the groups were compared, it was found that the surgical duration in the first and second set of 15 cases were significantly shorter in Group 2, mainly because of the familiarity of the otorhinolaryngologists with the anatomy of the region and the surgical equipment used. In the third set of 15 cases, the average surgical duration of both the groups became equal with an increase in the ophthalmologist’s endoscopic surgery experience level. When the learning curves were evaluated between two surgeons, it was observed that all parameters, except for the duration of surgery, were parallel with each other and that there were similar success and complication rates. In addition, after 30 cases, the surgical success rates reached the levels reported in the literature with surgical experience, and the complications were reduced to acceptable levels in accordance with the literature. The results obtained in this study are expected to guide surgeons who want to start this surgery. Furthermore, an important point that should be emphasized for new surgeons is the importance of undertaking surgical courses as well as video and cadaveric training in accordance with the guidelines defined by the Royal Collage of Ophthalmologists and The British Association of Otorhinolaryngology for Head and Neck Surgeons. It is also important to complete these trainings before surgery and accompany another surgeon experienced in this field during these operations.

In conclusion, after the necessary preliminary preparation are made for a surgery, EE-DCR can be applied with high success and low complication rates by otolaryngologists, by ophthalmologists not familiar with endoscopic surgery, and by surgeons after an experience of 30 cases.
